# Uptake of HIV/AIDS Services Following a Positive Self-Test Is Lower in Men Than Women in the Democratic Republic of the Congo

**DOI:** 10.3389/fmed.2021.667732

**Published:** 2021-07-30

**Authors:** Serge Tonen-Wolyec, Charles Kayembe Tshilumba, Salomon Batina-Agasa, Alliance Tagoto Tepungipame, Laurent Bélec

**Affiliations:** ^1^Ecole Doctorale Régionale d'Afrique Centrale en Infectiologie Tropicale, Franceville, Gabon; ^2^Faculty de Medicine, University de Bunia, Bunia, Democratic Republic of the Congo; ^3^Faculty of Medicine and Pharmacy, University of Kisangani, Kisangani, Democratic Republic of the Congo; ^4^Laboratory of Virology, Hôpital Européen Georges Pompidou, University de Paris, Paris Sorbonne Cité, Paris, France

**Keywords:** HIV, self-testing, linkage to care, men, Democratic Republic of the Congo

## Abstract

As far as HIV self-testing (HIVST) is concerned, proving the link to HIV care for users with a positive result contributes to understanding the implementation of HIVST. We sought to examine whether there were differences by sex in the uptake of HIV services following a positive self-test in the Democratic Republic of the Congo (DRC). This was a mixed-methods study exploring linkage to care for HIVST through a secondary analysis of collected data from three pilot surveys recently conducted in three cities (Kinshasa, Kisangani, and Kindu) during 2018 and 2020 in the DRC. Linkage to HIV care was defined as delayed when observed beyond 1 week. A total of 1,652 individuals were self-tested for HIV. Overall, the proportion of linkage to HIV care was high (*n* = 258; 82.2%) among individuals having a positive result with HIV self-test (*n* = 314), but it was significantly lower in men (65.2%) than women (89.2%). Furthermore, linkage to HIV care of men was significantly delayed as compared with that of women (40.0 vs. 20.7%). These findings show a lower uptake of care following a positive self-test in men than women. This trend already previously observed in sub-Saharan Africa shed light on the need to increase linkages to care among men newly diagnosed through HIV self-testing.

## Introduction

Diagnosing 95% of all people living with HIV (PLWHIV) is the first of three global 95–95–95 targets set by the UNAIDS to end the HIV epidemic by 2030 (1). Indeed, HIV testing is the principal gateway to HIV care and prevention services (2). In the Democratic Republic of the Congo (DRC), UNAIDS estimates that <60% of HIV-infected people know their seropositivity (3). Despite important progress in the scaling up of HIV testing in the DRC in the last 10 years, HIV testing remains remarkably deficient among men (4).

HIV self-testing (HIVST) is a new approach with the potential to increase uptake of HIV testing, especially among men, and other specific groups such as adolescents and key populations. The World Health Organization (WHO) defines the HIVST as a process in which an individual performs an HIV test and interprets the result, often in a private setting (5). Previous pilot studies have shown that the HIVST is practicable, acceptable, and accurate among the Congolese population (6–9). However, although the HIVST has the potential to reach men, the evidence on the linkage to care for users with a positive result remains to be established (10, 11). Furthermore, linkage to care after performing an HIV self-test is an additional concern because the HIVST can be performed through unassisted approaches in communities without proven monitoring and evaluation methods (10). The reasons explaining a low proportion of linkage to care among men in Africa may include the notorious ignorance of the benefits of antiretroviral therapy, the risk of losing one's job due to frequent clinic visits, the cost of transportation or fear of visibility as an HIV clinic client among asymptomatic patients, and death among those with AIDS (10–13).

With the current “test and treat” approach dictated by WHO since 2016 (14), barriers to assessing eligibility before initiation of antiretroviral therapy have been circumvented (15). Linkage to HIV care for HIVST is currently considered as accessing a healthcare provider through a clinic at four complementary different stages: the possibility to obtain accurate confirmation of HIV positivity, the enrolment into care after diagnosis, the onset of antiretroviral treatment, and the high adherence to antiretroviral treatment (2). However, evidence concerning the opportunity for HIV-positive individuals to receive post-test counseling and immediately enroll on HIV care for community-based unassisted HIVST remains unclear (10). Several studies have reported low proportion of linkage to care among men and have therefore explored other strategies such as home follow-up to improve linkage to care among men (11, 16, 17).

As far as HIVST is concerned, proving the link to care for users with a positive result contributes to understanding the implementation of HIVST in the DRC. In the present study, we sought to examine the linkage to care among Congolese previously self-tested in the three pilot surveys recently conducted in three main cities (Kinshasa, Kisangani, and Kindu) in the DRC.

## Materials and Methods

### Study Design

This was a mixed-methods study exploring linkage to care for HIVST through a secondary analysis of collected data from three pilot studies aimed at evaluating different distribution models of the HIVST during July 2018 and April 2020 in the DRC, followed by in-depth interviews. The STROBE (18) and COREQ (11) guidelines were followed for reporting quantitative and qualitative data, respectively.

### Study Setting

This multi-centric survey was carried out in the city of Kinshasa, the capital of the DRC; Kisangani, the capital city of the province of Tshopo; and Kindu, the capital city of the province of Maniema. These cities have different sociocultural and geographical contexts. A total of 18 study sites, integrating HIV testing and care settings, were selected for the study, including eight sites in Kinshasa (Marechal, Bomoi, Elonga, Kimia, Matonge, and Saint Joseph Health Centers; and Kalembelembe and Kimbondo Pediatric Hospitals), six sites in Kindu (Lumbulumbu, Kasuku-2, Sokolo, and Mikonde Health Centers; and Kindu and Alunguli General Referral Hospitals), and four sites in Kisangani (University hospital of Kisangani, Kabondo General Referral Hospital, and Neema and Saint-Joseph Health Centers).

### Study Population and Participant Recruitment

**Figure 1:**
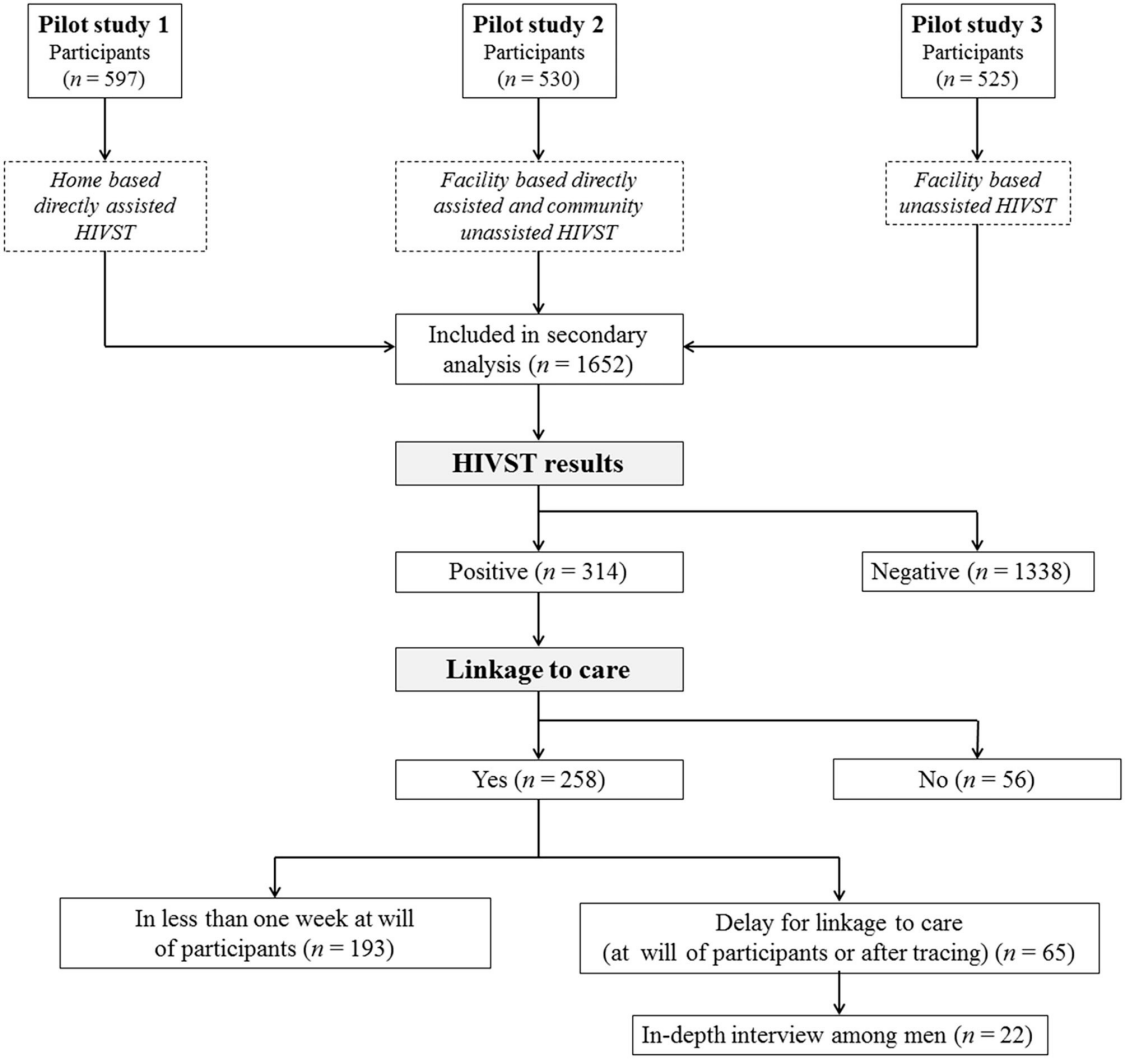
Flowchart showing the recruitment of the study participants in pilot studies 1, 2, and 3 and follow-up for linkage to care.

All participants were volunteers who were recruited from adolescents at home through a door-to-door approach (pilot study 1) and the general public at high risk for HIV infection (pilot studies 2 and 3), using different delivery HIVST approaches previously reported including home-based directly assisted HIVST by peer educators (pilot study 1) (7), facility-based directly assisted and community unassisted HIVST (pilot study 2) (9), and facility-based unassisted HIVST (pilot study 3) (8) (Figure 1). Eligible participants were between 15 and 49 years of age, were unaware of their HIV status, and were able to give written informed consent. Individuals on antiretroviral treatment or pre-exposure prophylaxis, transgenders, or persons who did not meet the study criteria were excluded.

### Sampling and Sample Size

Because this is secondary data analysis for the quantitative study, it was only possible to analyze the *post-hoc* power of the study. For the qualitative study, convenience sampling was used among the self-tested men with positive results who arrived in health facilities for linkage to care after 1 week.

### Data Collection and Study Procedure

The HIVST was performed using the blood-based self-test kit Exacto Test HIV (Biosynex, Strasbourg, France), as previously reported (6–9, 19).

The participants completed a self-administered baseline questionnaire to collect data on their demographic characteristics, sexual behavior, and HIV testing history, after which they received pretest HIV counseling. Pretest counseling consisted of a 30-min conversation: (i) to review the reasons why participants wanted to be tested for HIV; (ii) to learn about their knowledge of HIV prevention methods; (iii) to help them understand the consequences of HIV risk behaviors; (iv) to assess their level of risk; (v) to provide them with psychological support if their test result came back positive; and (vi) to advise them to be retested 3 months later if they had taken a risk. After HIVST in community or facility, participants who were identified as HIV positive were verbally advised to be link to HIV care at one of the 18 selected health facilities according to their preference. Linkage to care consisted of confirmation of positivity, post-test counseling, and initiation of antiretroviral treatment. Note that only delayed patients were traced, and the time frame for tracing HIV self-test users who tested positive but were not voluntarily linked to care was 2 weeks after HIVST. Men who linked to care after 1 week either voluntarily or by tracing were asked voluntarily to participate in in-depth interviews to understand the reasons for the low proportion and delay for linkage to care.

With a semi-structured guide containing open-ended questions, the in-depth interviews were conducted according to the phenomenological approach adapted to qualitative research, as described by Hsieh and Shannon (20). Note that phenomenology approach focuses on the commonality of a lived experience within a particular group (20). Thus, in this study, the lived experience was the linkage to care for HIVST, and the particular group was men. The questions were pretested with three men who had already used the HIVST kits for revising the study tool before actual data collection. Interviews were scheduled at the participant's convenience. Participants were contacted by telephone to arrange an appropriate time for the interview. Interviews were conducted in French, Lingala, or Swahili at the convenience of the participant. The average duration of these interviews was 45 min (minimum 35 min and maximum 60 min).

### Outcomes

The principal study outcomes were the linkage to care and the delay for linkage to care. Linkage to care was operationally defined as the reaching for HIV-positive confirmation, the receiving of post-test counseling, and the initiation of the antiretroviral treatment. The total follow-up time to evaluate linkage to care in this study was 30 days. Linkage to care was optimal when it was observed in less than a week, whereas it was considered delayed when observed beyond 1 week.

As previously reported, high risk for HIV infection was defined as a history of unprotected sex with one or more partners in the past 6 weeks as well as exposure to any of the following high-risk factors in the previous 6 months: multiple (i.e., >2) partners; homosexual intercourse (asked of men); receipt of gifts, cash, or other compensation in exchange for sex (asked of women); or infection with another sexually transmitted disease. Participants self-reported the information regarding the risk for HIV. Individuals exposed to any of these factors were classified as “high risk”; the remaining participants were classified as “low risk” if they did not report any sexual activity in the past 6 weeks and as “moderate risk” if otherwise (9, 21, 22). Educational level was categorized according to the educational system of the DRC, as follows: (i) low: unschooled or attending primary school; (ii) middle: attending college (training of 6 years) or technical school (training of 4 years); and (iii) high: attending bachelor's degree, graduate degree (training of 2 years after Bachelor's degree), or postgraduate degree, as previously reported (6).

### Ethical Considerations

Ethical approval for this survey was obtained from the Ethics Committee of the School of Public Health of the University of Kinshasa. Written informed consent was obtained from all participants. All participants with HIV-positive result for HIVST who were linked to care were provided with antiretroviral therapy and follow-up according to the DRC's national first-line therapeutic protocol including tenofovir, lamivudine, and dolutegravir, as recommended by the WHO (23).

### Data Management and Analysis

Overall linkage to care, delay for linkage to care, and strategy for improving linkage to care were evaluated quantitatively and/or qualitatively.

After identification and consolidation of the data for secondary data analysis from the three raw databases from the three pilot studies, continuous variables were expressed as means (±standard deviations) or median and interquartile range, as appropriate. Frequencies and proportions were used to describe categorical variables and were compared using Pearson's chi-squared test or Fisher's exact test, as appropriate. Unadjusted odds ratios of linkage to care and delay for linkage to care were estimated using the bivariate models. Multivariable-adjusted odds ratios of linkage to care and delay for linkage to care were estimated using the logistic regression analysis. Note that all factors (sociodemographics, sex behavior, and past story regarding HIV testing) studied were included in the bivariate models. However, only factors with *p* < 0.2 in the bivariate analysis were entered into the multivariate analysis. All quantitative analyses were performed using IBM SPSS Version 20 (Chicago, IL, USA) and XLSTAT (Addinsoft, Paris, France).

All qualitative data were first translated into French and then into English. Transcripts were analyzed through an inductive approach; thus, themes were identified during the course of analysis (24). In order to limit interviewers' biases due to preconceived ideas or theoretical perspectives when analyzing qualitative data, two different authors had independently analyzed the responses and coded them manually. Coding concepts were grouped into diverse categories and then linked and compared within inductive analysis (25). After the first list of thematic codes was generated, the answers were refined and grouped according to similarities (25).

## Results

### Study Participants

A total of 1,652 individuals who had performed the HIV self-test were eligible for secondary data analysis, including 597 adolescents from pilot study 1, 530 high-risk people from pilot study 2, and 525 high-risk people from pilot study 3 (Figure 1). After HIVST, 314 participants had reported a positive result and were followed up for linkage to care evaluation. The baseline characteristics of the total study participants and the participants with a positive self-test result are depicted in Table 1. In brief, among the participants included in the linkage to care assessment, 70.7% were female participants; 66.2% were aged between 15 and 24 years; 85.4% were single; 46.8% were students; and 40.8% were attending college or technical school. The majority of participants had never been tested for HIV (71.3%) and had no knowledge of the existence of HIVST (68.8%). The interview was conducted among 22 men who had been recruited among men who tested positive for HIV and who arrived after 1 week at the health facilities either voluntarily or by tracing for confirmation, post-test counseling, and treatment. Among them, the majority were <24 years old. Approximately three-quarters were single. One-third were students, one-third were employed or self-employed, and one-third were unemployed. Low educational level was observed in 22.7% of participants; a middle level was observed in 45.5%, and a high level was observed in 31.8% of participants.

**Table 1:** Characteristics of 1,652 participants using the HIV self-test and 314 participants with positive results.

**Characteristics**	**Total participants**	**Positive participants with self-test included in linkage to care analysis**
	**(*n* = 1,652)**	**(*n* = 314)**
	***n* (%)**	***n* (%)**
**Sex**		
Male	664 (40.2)	92 (29.3)
Female	988 (59.8)	222 (70.7)
**Age group**		
15–24 years	1,080 (65.4)	208 (66.2)
25–34 years	362 (21.9)	66 (21.0)
35–44 years	139 (8.4)	35 (11.1)
>44 years	71 (4.3)	5 (1.6)
**Partnership and civil status**		
Single	1,237 (74.9)	268 (85.4)
Married/partnered	415 (25.1)	46 (14.6)
**Occupation**		
Student	928 (56.2)	147 (46.8)
Employed	303 (18.3)	79 (25.2)
Unemployed	421 (25.5)	88 (28.0)
**Educational level^#^**		
Low	259 (15.7)	102 (32.5)
Moderate	904 (54.7)	128 (40.8)
High	489 (29.6)	84 (26.8)
**Risk of HIV infection^£^**		
Low risk	800 (28.4)	40 (12.7)
Moderate risk	359 (21.7)	69 (22.0)
High risk	493 (29.8)	205 (64.3)
**Previously tested for HIV**		
Never tested	1,007 (61.0)	224 (71.3)
Ever tested	645 (39.0)	90 (28.7)
**Previous knowledge about existence of HIV self-testing**		
Yes	467 (28.3)	98 (31.2)
No	1,185 (71.7)	216 (68.8)
**Previously self-tested for HIV**		
Never self-tested	1,578 (95.5)	312 (99.4)
Ever self-tested	74 (4.5)	2 (0.6)

# *Educational level was categorized according to the educational system of the Democratic Republic of the Congo, as follows: (i) low: unschooled or attending primary school; (ii) middle: attending college (training of 6 years) or technical school (training of four years); and (iii) high: attending bachelor's degree, graduate degree (training of 2 years after Bachelor's degree), or postgraduate degree, as previously reported (6)*.

£ *High risk for HIV infection was defined as a history of unprotected sex with one or more partners in the past 6 weeks as well as exposure to any of the following high-risk factors in the previous 6 months: multiple (i.e., >2) partners; homosexual intercourse (asked of men); receipt of gifts, cash, or other compensation in exchange for sex (asked of women); or infection with another sexually transmitted disease. Individuals exposed to any of these factors were classified as “high risk”; the remaining participants were classified as “low risk” if they did not report any sexual activity in the past 6 weeks and as “moderate risk” if otherwise (9, 21, 22)*.

### Overall Linkage to Care

Among 314 participants having a positive result with HIV self-test, 258 had completed the linkage to care assessment, yielding an overall proportion of linkage to care for HIVST at 82.2%. The linkage to care was in <1 week at the will of participants in 74.8% of cases (Figure 1). Overall, the mean time for linkage to care was 9.7 ± 2.4 days. However, it was 5.4 ± 1.2 days among participants linked to care in less than a week and 21.1 ± 5.7 days among latecomers. The variables “sex,” “age group,” and “educational level” were significantly associated with linkage to care in bivariate models. Indeed, the linkage to care for HIVST was significantly low among men than women (65.2 vs. 89.2%; crude OR: 0.2 [95% CI: 0.1–0.4]) and participants with high educational level compared with those with low educational level (72.6 vs. 87.3%; crude OR: 0.4 [95% CI: 0.2–0.8]). However, the linkage to care for HIVST was significantly high among participants aged between 15 and 24 years (84.6 vs. 69.7%; crude OR: 2.4 [95% CI: 1.3–4.6]) and those aged between 35 and 44 years (97.1 vs. 69.7%; crude OR: 14.8 [95% CI: 1.9–115.6]) compared with those aged between 25 and 34 years. Other variables such as “risk of HIV infection” and “past HIV testing” had a *p* < 0.2 in the bivariate analysis.

As shown in Table 2, multivariate analysis showed that male gender (adjusted OR: 0.7, 95% CI: 0.5–0.9) was significantly associated with the decrease of the linkage to care, whereas the proportion of linkage to care was increased among young participants (adjusted OR: 2.0, 95% CI: 1.0–4.0) and participants aged 35–44 years (adjusted OR: 2.8, 95% CI: 1.2–4.7) compared with those aged 25–34 years.

**Table 2:** Factors associated with linkage to care among 314 participants interpreting their self-test results as positive and to delay for linkage to care among 258 participants linked to care.

	**Linkage to care**			**Delay for linkage to care**		
	**(** ***n*** **= 314)**			**(** ***n*** **= 258)**		
	**Yes**	**No**	**aOR**	**Yes**	**No**	**aOR**
	***n* = 258**	***n* = 56**	**(95% CI)**	***n* = 65**	***n* = 193**	**(95% CI)**
	***n* (%)**	***n* (%)**		***n* (%)**	***n* (%)**	
**Sex**						
Male	60 (65.2)	32 (34.8)	0.7 (0.5–0.9)	24 (40.0)	36 (60.0)	1.8 (1.1–2.7)
Female	198 (89.2)	24 (10.8)	1	41 (20.7)	157 (79.3)	1
**Age group**						
15–24 years	176 (84.6)	32 (15.4)	2.0 (1.0–4.0)	41 (23.3)	135 (76.7)	NA
25–34 years	46 (69.7)	20 (30.3)	1	12 (26.1)	34 (73.9)	NA
35–44 years	34 (97.1)	1 (2.9)	2.8 (1.2–4.7)	10 (29.4)	24 (70.6)	NA
>44 years	2 (40.0)	3 (60.0)	0.7 (0.3–22.1)	2 (100)	0 (0)	NA
**Educational level^#^**						
Low	89 (87.3)	13 (12.7)	1	23 (25.8)	66 (74.2)	NA
Moderate	108 (84.4)	20 (15.6)	0.9 (0.4–4.6)	31 (28.7)	77 (71.3)	NA
High	61 (72.6)	23 (27.4)	0.7 (0.5–1.1)	11 (18.0)	50 (82.0)	NA
**Risk of HIV infection^£^**						
Low risk	32 (80.0)	8 (20.0)	1	3 (9.4)	29 (90.6)	1
Moderate risk	56 (81.2)	13 (18.8)	1.2 (0.7–4.9)	8 (14.3)	48 (85.7)	1.5 (0.7–4.1)
High risk	170 (82.9)	35 (17.1)	1.4 (0.6–6.6)	54 (31.8)	116 (68.2)	2.1 (1.2–3.8)
**Previously tested for HIV**						
Never tested	188 (83.9)	36 (16.1)	1.1 (0.8–7.6)	45 (23.9)	143 (76.1)	NA
Ever tested	70 (77.8)	20 (22.2)	1	20 (28.6)	50 (71.4)	NA

*aOR, adjusted odds ratio; CI, confidence interval; n, number; NA, not applicable; NS, not significant*.

* *p-value calculated using regression analysis*.

# *Educational level was categorized according to the educational system of the Democratic Republic of the Congo, as follows: (i) low: unschooled or attending primary school; (ii) middle: attending college (training of 6 years) or technical school (training of four years); and (iii) high: attending bachelor's degree, graduate degree (training of 2 years after bachelor's degree), or postgraduate degree, as previously reported (6)*.

£ *High risk for HIV infection was defined as a history of unprotected sex with one or more partners in the past 6 weeks as well as exposure to any of the following high-risk factors in the previous 6 months: multiple (i.e., >2) partners; homosexual intercourse (asked of men); receipt of gifts, cash, or other compensation in exchange for sex (asked of women); or infection with another sexually transmitted disease. Individuals exposed to any of these factors were classified as “high risk”; the remaining participants were classified as “low risk” if they did not report any sexual activity in the past 6 weeks and as “moderate risk” if otherwise (9, 21, 22)*.

Qualitative observations provided additional insights into the factors that influenced linkage to care. When participants were asked to provide reasons for lack of linkage to care, their responses emphasized fear of the unknown, fear of stigma, and doubt about the result of self-testing:

“I was afraid to confirm my positive result because if I do, I will be a candidate for death while I am still young and have family responsibilities. I preferred to remain in ignorance of my HIV status.” (Interview, 23-year-old man, Kindu).

“I knew the risk I had taken in the past, so I found it unnecessary to confirm my HIV status because I knew I was infected.” (Interview, 34-year-old man, Kinshasa).

“I was afraid of stigma because at the hospital sometimes people's HIV status is known to everyone…” (Interview, 45-year-old man, Kisangani).

“I doubted the result of the self-test because I was not sick and I had no signs of HIV infection…” (Interview, 38-year-old man, Kinshasa).

### Delay for Linkage to Care

The delay for linkage to care was observed among 65 (25.2%) participants (Figure 1). The variables “sex” and “risk of HIV infection” were significantly associated with delay for linkage to care in bivariate models. No other variables had a *p* < 0.2 in the bivariate analysis. In the multivariate model, the delays for linkage to care were significantly high among men (40.0 vs. 20.7%; adjusted OR: 1.8 [95% CI: 1.1–2.7]) and users with a high risk of HIV infection (31.8 vs. 9.4%; adjusted OR: 2.1 [95% CI: 1.2–3.8]) compared with women and users with low risk of HIV infection, respectively (Table 2).

Of note, no differences were observed when comparing linkage to care and delay for linkage to care in the unassisted vs. the directly assisted HIVST.

## Discussion

We herein report on linkage to care among individuals previously self-tested in the three pilot surveys recently conducted in three cities (Kinshasa, Kisangani, and Kindu) in the DRC. Overall, the proportion of linkage to care was high (82.2%) among individuals having a positive result with HIV self-test, and but it was significantly lower in men (65.2%) than women (89.2%). Furthermore, linkage to care of men was significantly delayed as compared with that of women (40.0 vs. 20.7% of cases). Taken together, these findings suggest in the DRC the trend already previously observed in sub-Saharan Africa of a lower uptake of care following a positive HIV self-test in men.

HIVST is a promising approach to reach populations far beyond traditional HIV testing, such as men (5). Furthermore, one of the many hypotheses of transmission in the DRC is based on the belief that mature men, often over the age of 40, who have been infected for several years, are the main vectors of transmission via their numerous sexual partners, often adolescent girls (4). Interventions aiming to increase HIV testing among men and linking them to care are very important in the HIV response in DRC and sub-Saharan Africa.

Linkage to care is a critical step following HIVST to ensure that those who test positive confirm their HIV status and receive counseling and initiation of antiretroviral therapy when their status is confirmed (16, 26). However, several authors have debated the definition of what should be considered delayed uptake of care. For the Centers for Disease Control and Prevention (CDC) experts, linkage to care within 1 week can be considered optimal behavior (27). However, for Njau and colleagues, even a 3-month follow-up delay would not be sufficient to assess linkage to care (28). Because it is possible that people who test positive, but are not immediately cared for, may take some time to change their behavior. This debate about the delay to consider when assessing linkage to care would directly impact our results because it would overestimate the proportion of linkage to care and underestimate the proportion of delayed linkage to care. It is within this framework that the qualitative approach has allowed us to understand the gaps in linkage to care among men. In our study, one participant gave this reason for delaying linkage to care as follows: “I needed some time to adopt a new behavior because I need to put my life in order.”

The majority of study participants (82.2%) with a positive HIV self-test result were linked to care with an optimal linkage to care proportion of 74.8% (<1 week). These proportions of linkage to care are lower than those previously reported by Chipungu and colleagues in a representative cross-sectional survey at Lusaka, Zambia, in which intention to link to care after a positive result with HIVST was 90% (10).

Comparing linkage to care among men who tested in the hospital vs. HIV self-tested men, Korte and colleagues reported in Uganda that men who tested positive through self-testing may not be as likely to link to care as men who tested positive at a clinic (17). This question of linkage to care deserves to be studied in more depth for a better understanding in the long term. Currently, large-scale studies funded by PEPFAR and the Global Fund to Fight HIV, Tuberculosis, and Malaria are underway in the DRC to assess the issue of linkage to care for HIVST in the Congolese context.

Innovative strategies are important to promote linkage to care among men. The HIV assisted partner services have been recommended as a strategy to increase HIV case finding. However, a pilot study carried out in Kenya reported a low rate of linkage to care (only two-thirds) among index clients and sex partners (16). Offering home follow-up for initiation of antiretroviral therapy is an option to bridge the linkage to care gap at the clinic (11). This option merits exploration through operational research, as one of study participants responded that: “I prefer that the confirmatory test be performed at home and that the treatment also be delivered at home or privately if possible.”

## Strength and Limitations

One strength of the study is the inclusion of participants living in the DRC, not previously studied, the largest French-speaking country in sub-Saharan Africa, which gives a certain impact to our results particularly in the cultural context of Central Africa. This study has some limitations. First, linkage to care is expressed as an intention and is not measured as an actual behavior. This is because the study was a feasibility study conducted prior to the introduction of the HIV self-test in the DRC. Intention to link to care may not translate into actual linkage to care behavior. Thus, there is a need to evaluate linkage to care when the self-test will be effectively rolled out in the DRC. Furthermore, the study population was from three cities of the DRC only and is not representative of the entire country. With the follow-up time to evaluate linkage to care of only 30 days, this study may underestimate the overall linkage to care proportion. The sample size was furthermore limited. Lastly, we did not conduct in-depth interviews with those not willing to link to care within a week.

In conclusion, this study shows an overall high proportion of linkage to care among individuals having a positive result with HIV self-test. However, men were less linked to care and linked to care late comparatively than women. These findings highlight the need to implement innovative strategies for increasing the linkage to care, especially in men living in the DRC.

## Data Availability Statement

The raw data supporting the conclusions of this article will be made available by the authors, without undue reservation.

## Ethics Statement

The studies involving human participants were reviewed and approved by Commité d'éthique de l'Ecole de Santé Publique de l'Université de Kinshasa. Written informed consent to participate in this study was provided by the participants' legal guardian/next of kin.

## Author Contributions

ST-W and LB conceived and designed the research. ST-W and AT were involved in volunteer recruitment and follow-up. ST-W, SB-A, and LB performed statistical analyses. ST-W, CK, and LB analyzed the results and drafted the manuscript. All authors contributed to the article and approved the submitted version.

## Conflict of Interest

The authors declare that the research was conducted in the absence of any commercial or financial relationships that could be construed as a potential conflict of interest.

## Publisher's Note

All claims expressed in this article are solely those of the authors and do not necessarily represent those of their affiliated organizations, or those of the publisher, the editors and the reviewers. Any product that may be evaluated in this article, or claim that may be made by its manufacturer, is not guaranteed or endorsed by the publisher.
